# Single Enema Fecal Microbiota Transplantation in Cats With Chronic Enteropathy

**DOI:** 10.1111/jvim.70054

**Published:** 2025-04-10

**Authors:** Dimitra A. Karra, Jan S. Suchodolski, Shelley J. Newman, Evgenia Flouraki, Jonathan A. Lidbury, Joerg M. Steiner, Panagiotis G. Xenoulis

**Affiliations:** ^1^ Clinic of Medicine, Faculty of Veterinary Science University of Thessaly Karditsa Greece; ^2^ Gastrointestinal Laboratory, Department of Small Animal Clinical Sciences Texas A&M University Texas USA; ^3^ Newman Specialty VetPath New York USA; ^4^ Clinic of Surgery, Faculty of Veterinary Science University of Thessaly Karditsa Greece

**Keywords:** CIE, feline, FMT, lymphoma

## Abstract

**Background:**

Chronic enteropathies (CE) are common in cats, and alterations of the intestinal microbiota might be involved in the pathogenesis.

**Hypothesis/Objectives:**

To evaluate the efficacy of a single enema fecal microbiota transplantation (FMT) in improving intestinal dysbiosis and clinical scores in cats with CE.

**Animals:**

Twenty‐eight cats with either chronic inflammatory enteropathy (CIE; *n* = 19) or small cell gastrointestinal lymphoma (SCGL; *n* = 9) were prospectively enrolled.

**Methods:**

Eleven cats were randomly selected to receive a single enema FMT (FMT‐group), and 17 cats were used as controls. Clinical activity was determined using the Feline Chronic Enteropathy Activity Index (FCEAI), and intestinal dysbiosis was determined using the feline dysbiosis index (DI) on the day of FMT (T0) and 30 days after FMT (T1).

**Results:**

At T0, 14/28 cats had an abnormal DI > 0. No significant difference was found in the DI from T0 to T1 in the FMT group (mean[SD]: 0.01[2.5] vs. 0.7[2.1]; *p* = 0.47). No significant difference was found in the DI between the FMT group and the control group at T1 (mean[SD]: −0.7[2.1] vs. 0.8[1.8]; *p* = 0.92). FCEAI significantly decreased at T1 compared to T0 in the FMT group (median[IQR] 10.0[7.7–11.3] vs. 4.5[4–5]; *p* = 0.002). No significant difference was found in the FCEAI between the FMT group and the control group at T1 (median[IQR] 4.5[4–5] vs. 4[3–5.75]; *p* = 0.64).

**Conclusions:**

In this study, single enema FMT did not lead to a significant improvement in DI or FCEAI in cats with CE compared to controls.

**Abbreviations**
ARE: antibiotic‐responsive enteropathy
CE: chronic enteropathy
CIE: chronic inflammatory enteropathy
FCEAI: feline chronic enteropathy activity index
fPLI: feline pancreatic lipase immunoreactivity
FRE: food‐responsive enteropathy
fTLI: feline trypsin like immunoreactivity
GI: gastrointestinal
SCGL: small cell GI lymphoma
Spec fPL: feline‐specific pancreatic lipase

## 1 Introduction

Chronic enteropathy (CE) describes a diverse group of gastrointestinal (GI) diseases in cats [1]. CE has been categorized into chronic inflammatory enteropathy (CIE) and small cell GI lymphoma (SCGL) [1, 2, 3, 4, 5, 6]. Subclassification of CIE is based on the response to treatment into immunosuppressant‐responsive enteropathy (IRE) and food‐responsive enteropathy (FRE) [1]. Studies in humans, dogs, and cats suggest that intestinal dysbiosis has a great impact on the pathogenesis and progression of GI diseases [7]. A large subset of dogs with chronic GI diseases has dysbiosis [8]. In another recent study, 64% of the CE dogs have an abnormal DI [9]. In cats, approximately 76% of cats with CE have an altered fecal microbiota, based on DI [10], and cats with CE have patterns of dysbiosis similar to those found in humans with IBD [11].

Therapeutic approaches targeting dysbiosis may play an important role in complex diseases such as CE in cats. Current therapeutic methods include dietary modification, antibiotic administration, immunosuppressive therapy, or a combination of these. Corticosteroids are the mainstay of immunosuppressive treatment [12, 13], although they have no direct effect on the composition of the fecal microbiome and were only associated with partial [14] or no restoration [15, 16] of normobiosis in dogs with CE. A possible therapeutic option for rectification of dysbiosis is fecal microbiota transplantation (FMT).

FMT is the administration of feces from a healthy donor into the gut of a diseased recipient with the aim to modulate or replace the recipient's intestinal microbiota and decrease disease activity [17]. In humans, FMT is an effective treatment for recurrent *Clostridioides difficile* infection [18, 19] and has also been evaluated as a treatment for IBD and IBS [20, 21, 22]. In humans with IBD using a multi‐donor repeated FMT (i.e., enemas 5 days/week for 8 weeks) a higher percentage of patients treated with FMT achieved the primary outcome of steroid‐free clinical remission compared with the placebo group. During this study, the administration of specific drugs was allowed as long as the dose was stable for 4 weeks [23]. In 24 studies in humans with IBD that received FMT with or without steroids, 33% of patients achieved clinical remission and 52% achieved a clinical response, with greater response rates if multiple FMT infusions were administered [24].

FMT in cats with CE has been described in a few case reports and one recent prospective study. In a case report, οne cat with a 16‐month duration of signs of GI disease achieved a 3‐month remission with one enema of fresh feces [25]. In a study, FMT capsules were given orally to cats with signs of GI disease and, according to their owners, 77% of cats showed clinical improvement, and 23% exhibited either no change or a worsening of their clinical signs [26]. Prospective blinded clinical trials are needed to assess the efficacy of FMT in cats with CE.

The aim of this study was to evaluate the efficacy of single enema FMT in cats with CE.

## 2 Materials and Methods

### 2.1 Ethics Approval

The study protocol was reviewed and approved by the Animal Ethics Committee of the University of Thessaly, Greece (AUP number:115/6.10.2020). The owners of each cat enrolled in the study signed an informed owner consent form before enrollment.

### 2.2 Study Sample

Cats presenting from September 2020 to October 2022 to the Clinic of Medicine of the University of Thessaly and to the referral hospital Animal Medical Center in Athens, Greece, for persistent or intermittent signs of GI disease of at least 3 weeks duration were considered for inclusion in the study. Cats were included in the study if (1) they had clinical signs compatible with CE (i.e., diarrhea or loose stools of > 3 weeks duration; vomiting ≥ 2 times per month for ≥ 2 months, weight loss, hyporexia, and/or anorexia); (2) had no other diseases that could explain the cat's clinical signs; and (3) had no history of administration of antibiotics, corticosteroids, or other immunosuppressive agents for at least 3 weeks prior to presentation. However, cats with a clinical presentation compatible with CE that had concurrent diseases that were well controlled with appropriate treatment were eligible for enrollment.

### 2.3 Study Design and Fecal Collection

All cats with CE underwent the same diagnostic investigations, consisting of a complete clinical history, physical examination, a complete blood count (CBC), serum biochemistry profile with electrolytes, serum concentrations of cobalamin, folate, feline pancreatic lipase immunoreactivity (measured as Spec fPL, IDEXX) [27] and feline trypsin‐like immunoreactivity (fTLI) [28], urinalysis, fecal parasitology using saturated zinc sulfate flotation, abdominal ultrasound, PCR for *Tritrichomonas foetus*, upper and lower GI endoscopy, and biopsy collection for histopathology and immunochemistry. The feline chronic enteropathy activity index (FCEAI) [29] was calculated for all cats with CE. A quantitative PCR (qPCR) based dysbiosis index (DI) for analyzing the fecal microbiota of cats was used [10]. Reexamination along with CBC, serum biochemistry profile with electrolytes, serum concentrations of cobalamin, folate, Spec fPL, and fTLI were repeated on day 30 after endoscopy.

Endoscopy was performed using a flexible videoendoscope (Pentax EG2490Κ, PENTAX Medical EMEA) with an outer diameter of 8 mm and a 2.4 mm biopsy channel. Biopsies were obtained using 2.2 mm forceps. Prior to endoscopy, food was withheld for 12 h, and 4–5 warm water enemas were performed to clean the colon while the cat was under general anesthesia. Feces were collected at baseline (T0; prior to endoscopy), naturally passed or passed during the enema, and at day 30 (T1), naturally passed after endoscopy. All fecal samples were placed in Eppendorf tubes and stored at −80°C until analyses.

CBC, biochemistry profile with electrolytes was performed using the same in‐house analyzers (Vetscan HM5 Hematology Analyzer and Vetscan VS2 Chemistry Analyzer). Serum concentrations of cobalamin, folate, Spec fPL, and fTLI were measured at the Gastrointestinal Laboratory at the Texas A&M School of Veterinary Medicine & Biomedical Sciences (VMBS).

### 2.4 Histopathology and Immunohistochemistry

Formalin‐fixed, paraffin‐embedded endoscopic biopsy samples were routinely stained with hematoxylin and eosin (H&E) for histopathologic examination. All the endoscopic biopsy samples were examined by a board‐certified veterinary anatomic pathologist, with special expertise in small animal GI histopathology. Findings were reported descriptively and numerically scored according to the WSAVA histopathologic scoring system [30]. Both inflammatory (i.e., presence of lymphocytes, plasma cells, eosinophils, neutrophils, and macrophages in the lamina propria) as well as morphological features (e.g., surface epithelial injury, crypt lesions such as dilatation, distortion, or hyperplasia, atrophy and fibrosis) were assessed histologically and assigned a score (normal = 0, mild = 1, moderate = 2, and marked = 3). Immunohistochemistry was conducted using a stepwise approach. Staining for T‐ and B‐cell markers (CD3, CD79a respectively) was performed if deemed necessary based on the pathologist's discretion depending on the results of H&E staining (i.e., number, size, and distribution of mucosal lymphocytes). Based on pathologist's discretion, clonality testing (PARR) was also performed.

### 2.5 Donor Cat Characteristics

Three unrelated donors were used, and a blend of equal amounts of each donor's fecal matter was used for the transplants [24]. Donors were between 2 and 7 years of age, client‐owned, indoor, fully vaccinated, dewormed, non‐obese, and clinically healthy for at least 1 year based on history (i.e., no episodes of vomiting and/or diarrhea, normal body condition scoring, and were fed a diet following the AAFCO criteria). Donors had not received any treatment known to affect gastrointestinal microbiota, including antibiotics, probiotics, or prebiotics for at least a year prior to fecal sample collection. Donor cats had a normal DI (DI < 0), normal CBC, serum biochemistry profile, and urinalysis; they were negative for feline leukemia virus and feline immunodeficiency virus. All three donors were PCR negative for *Clostridium perfringens* enterotoxin gene, net F toxin gene‐ *C. perfringens* , *C. difficile* , *Campylobacter jejuni* and *E. coli* , feline panleukopenia virus (FPV), *Salmonella* spp., *Tritrichomonas foetus*, and IFA testing for *Giardia* and *Cryptosporidium*.

### 2.6 FMT Protocol

Naturally passed feces were collected from each donor cat from the litter box. FMT infusions were constituted from the blended homogenized feces of the 3 unrelated cats to avoid a potential negative single donor effect [24]. Only fresh feces obtained within 12 h of defecation were used for the preparation of the transplants. Approximately 2 g/kg feces were dissolved in 0.9% sterile saline at a 1:3 ratio until homogenization was achieved [17]. Fecal particles that could not be mixed were removed. A FMT of 8 mL/kg of the fecal suspension was administered to each recipient cat via enema [17]. The appropriate volume of the fecal suspension was picked up through a 60 mL syringe, and the syringe was attached to a urethral catheter 2.7 mm [17].

FMT was performed after the completion of colonoscopy and biopsy collection, and FMT transplants were administered in the form of enemas. During the procedure, recipients remained under general anesthesia and in left lateral recumbency, and their caudal body was elevated approximately 30° from the surface. With the urinary catheter inserted by about 10 cm, the suspension was slowly administered in the proximal part of the rectum. Then, recipients were placed in sternal and left lateral recumbency for 10 min in each position to allow uniform distribution of the transplant. After the completion of the FMT procedure, cats were monitored to ensure a safe recovery from anesthesia. After endoscopy, all owners from both groups were asked to closely monitor each cat for aggravation of signs of GI disease or for new signs and report those to us to ensure that no adverse effects of the FMT were noticed.

Although our study is technically controlled, enemas without the addition of fecal material were not given to the control cats, and therefore it cannot be considered a placebo‐controlled study.

### 2.7 Quantitative Polymerase Chain Reaction (qPCR) Analysis

Total fecal DNA was extracted using the QIAamp PowerFecal Pro‐DNA Kit (QIAGEN) and an automatic extraction system (Thermo KingFisher Flex Magnetic Particle Purification 96 PCR Isolation System), according to the manufacturers' instructions. The qPCR assays were performed as previously reported [31, 32]. Briefly, the DNA concentration of the extract was measured by a spectrophotometer (NanoDrop 1000; Thermo Scientific) and normalized to 5 ng/μL. A mixture of 2 μL normalized DNA extract (5 ng/μl), 5 μL SsoFast EvaGreen supermix (Bio‐Rad Laboratories), 0.4 μL forward primer (400 nM), 0.4 μL reverse primer (400 nM), and 2.2 μL DNA‐free water was used for qPCR assays using a Bio‐Rad C1000 Touch Thermal Cycler (Bio‐Rad Laboratories). The protocol for the thermal cycler was as follows: initial denaturation at 98°C for 2 mins; 35 cycles with denaturation at 98°C for 3 s; and annealing for 3 s. All samples were analyzed in duplicate fashion, and the average of the two results was used for further analysis. The Bio‐Rad CFX Maestro 1.1 software (Bio‐ Rad Laboratories) was applied to analyze the qPCR results.

### 2.8 Clinical Activity Index

The clinical activity before and after treatment was evaluated using the FCEAI [29]. The FCEAI is based on five clinical criteria (each scored on a scale of 0–3: attitude/activity, appetite, vomiting, stool consistency, and weight loss) and the evaluation of three biochemical parameters (each scored with 1: increased total protein, increased ALT or ALP and decreased phosphorus). The total composite scores are evaluated as follows: 0–3, clinically insignificant; 4–5, mild; 6–8, moderate; 9 or higher, severe. The criterion “endoscopic lesions” was omitted from the calculation of the FCEAI because cats did not undergo repeated endoscopy evaluation after FMT [33, 34]. Owners and the clinician who did the reexamination of the cats were blinded to whether the cat had received FMT.

### 2.9 Treatments in Cats With CE

All cats received the same preventative antiparasitic treatment (Broadline, Boehringer Ingelheim) for the duration of the study. After endoscopy and biopsy collection, owners were advised to feed their cats the same hydrolyzed protein diet (Anallergenic, Royal Canin). In addition, all cats received cobalamin (250 μg/cat SQ every 2 weeks) for the duration of the study. Cats that did not respond to the diet or cats that refused to consume the diet were treated with prednisolone (starting dose 2 mg/kg, once daily, with gradual tapering), while cats with a diagnosis of SCGL were treated with prednisolone (same dosing protocol as for cats with CIE) plus chlorambucil (2 mg/cat every 2nd or 3rd day). Immunomodulatory treatment was started 14 to 30 days after endoscopy in all cats depending on the severity of the disease and the time needed for histopathology results to become available.

### 2.10 Statistical Analysis

Normality was tested using the Shapiro–Wilk test. Normally distributed data were compared using *t*‐tests. Mann–Whitney tests were used to compare non‐normally distributed data. DI was compared between groups at baseline using a Mann–Whitney test. DI and FCEAI before and after FMT were compared using a Wilcoxon test. Significance was set at *p* < 0.05. All data were analyzed using a commercially available statistical software package (GraphPad Prism 8).

## 3 Results

### 3.1 Signalment

A total of 26 cats with CE were included in the study. Of these, 19 were DSH, 3 DLH, 1 Norwegian Forest, 1 Bengal, 1 Birman, and 1 Persian. Cats with CE had a mean body weight of 4.9 kg (SD 1.5), mean BCS of 5.19 (SD 1.85) on a 9‐point scale, and a mean age of 10.77 years (SD 2.76). Sixteen cats were male (15 neutered) and 10 cats were spayed females.

### 3.2 Final Diagnoses

Based on clinical response, histopathology, and immunochemistry results, 18 cats were diagnosed with CIE (13 IRE 5 with FRE), and 8 with SCGL (Table 1).

**TABLE 1:** Results of qPCR for each individual taxa.

Variable	CE (*n* = 26)	CIE (*n* = 18)	SCGL (*n* = 8)
Bacteroides	5.39 (1.44–7.28)	5.52 (1.46–7.28)	5.34 (3.44–6.76)
		*p* = 0.59	
Bifidobacterium	5.51 (1.23–8.7)	5.62 (1.22–8.7)	5.14 (4.24–6.73)
		*p* = 0.41	
C. hiranonsis	6.19 (0.1–7.35)	6.4 (1.26–7.36)	5.9 (0.1–6.88)
		p = 0.03	
*E. coli*	4.84 (1.38–7.94)	4.61 (1.38–7.94)	3.98 (2–6.49)
		*p* = 0.73	
Faecalibacterium	5.42 (1.39–7.47)	5.19 (1.39–7.46)	6.26 (3.54–6.62)
		*p* = 0.43	
Streptococcus	3.66 (0.92–9.31)	3.6 (0.92–6.1)	3.79 (2.79–9.31)
		*p* = 0.77	
Turicibacter	5.43 (2.38–8.54)	5.46 (2.38–8.24)	5.28 (4.31–8.54)
		*p* = 0.89	
DI	0.61 (−4.2–3.52)	−0.21 (−4.2–3.22)	0.82 (−3.91–3.52)
		p = 0.5	

*Note:* Data expressed as median (IQR) log DNA/g of feces. Significance was set at *p* = 0.05.

### 3.3 Concurrent Diseases

Four cats had concurrent pancreatitis, two had diabetes mellitus, two had stable International Renal Interest Society (IRIS) stage II chronic kidney disease (CKD), two had stomatitis, one had hyperthyroidism, and one had idiopathic hypertension. All concurrent diseases of the cats were well controlled before and during the study period.

### 3.4 FMT, Treatment, and Follow‐Up

Of the 26 cats with CE included in the study, 10 were randomly selected to receive one FMT via enema (FMT‐group) on the day of the endoscopy (T0), while the remaining 16 cats did not receive FMT (non‐FMT group). Owners and the veterinarian who were responsible for the reexamination of each cat were blinded to the group to which the cat belonged to. At reexamination, feces were collected (T1) from all cats.

All cats were attempted to be switched to the same hydrolyzed protein diet after endoscopy (Anallergenic, Royal Canin). However, only 13 out of 26 cats actually consumed the diet exclusively, while one cat (Cat 9) consumed Anallergenic together with Hypoallergenic (Royal Canin) wet food. In one cat (Cat 5), the owner reported that clinical signs worsened with Anallergenic and switched to the previous diet, while in another cat (Cat 17) the diet was not changed due to the risk of dysregulation of the cats' diabetes mellitus that was well controlled with diet and insulin. The remaining 10 continued to consume their previous diet due to their refusal to consume the study protein diet.

### 3.5 Dysbiosis Index in Cats With CE at Baseline

**FIGURE 1:**
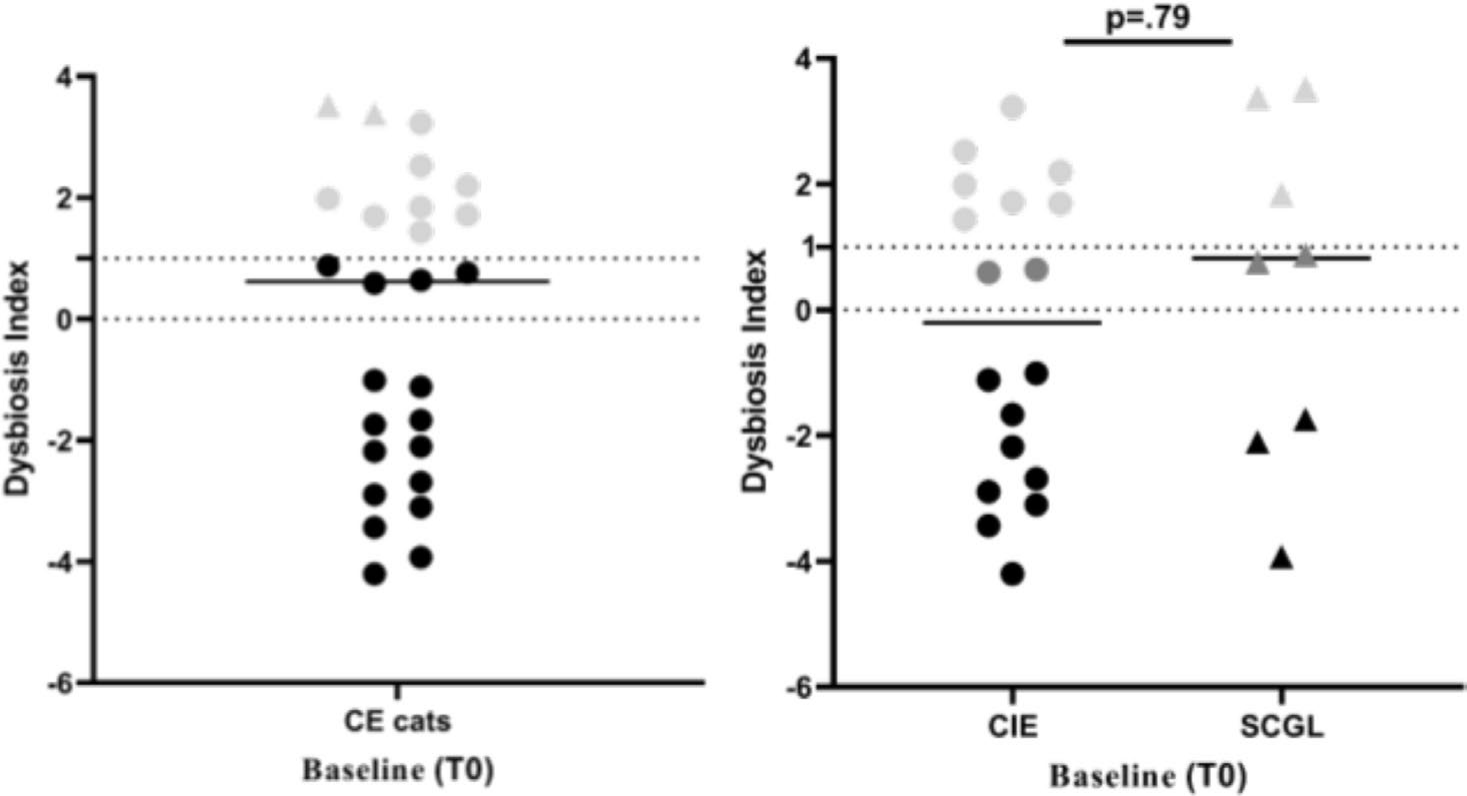
DI in cats with CE and in cats with CIE and SCGL at baseline (T0). No significant difference was found between cats with CIE and cats with SCGL at T0. Black symbols represent healthy microbiota (DI < 0), gray symbols represent mild dysbiosis (DI 0–1), and light gray symbols represent severe dysbiosis (DI > 1).

**FIGURE 2:**
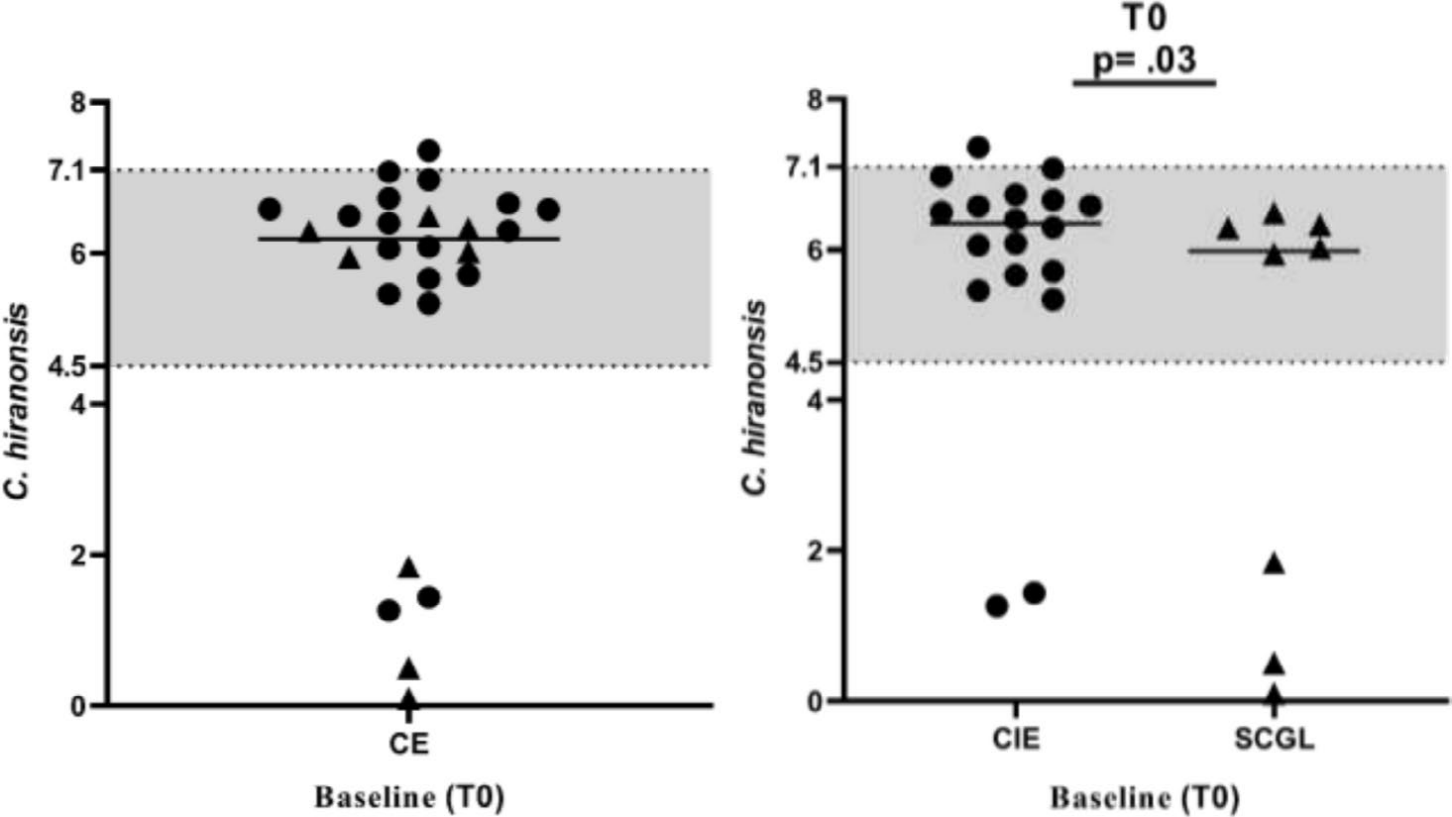
*C. hiranonsis* at T0 in cats with CE and in cats CIE and with SCGL. Cats with SCGL had a significantly lower abundance of *C. hiranonsis* (*p* = 0.03) than cats with CIE. Gray shading represents the normal abundance of *C. hiranonsis* (4.5–7.1). ⚫ Cats with diagnosis of CIE, ▲Cats with diagnosis of SCGL.

Quantitative real‐time PCR for *Bacteroides*, *Bifidobacterium*, *C. hiranonsis*, *E. coli* , *Faecalibacterium*, *Streptococcus*, and *Turicibacter* was included in the DI. At T0, 14/26 cats had dysbiosis (Figure 1). Of those, 4 had a mildly increased DI (0–1), while the remaining 10 had a more significantly increased DI (> 1). *C. hiranonsis* was below the lower limit of the reference interval (4.5–7.1) in 5/26 cats (Figure 2). Cats with SCGL had a significantly lower abundance of *C. hiranonsis* than cats with CIE (median 6.4 IQR 5.7–7.4 vs. median 5.9, IQR 1.2–6.3; *p* = 0.03) (Figure 2). Changes in each bacterial taxa are displayed in Table 1.

### 3.6 Dysbiosis Index in Cats With CE Before and After FMT

**FIGURE 3:**
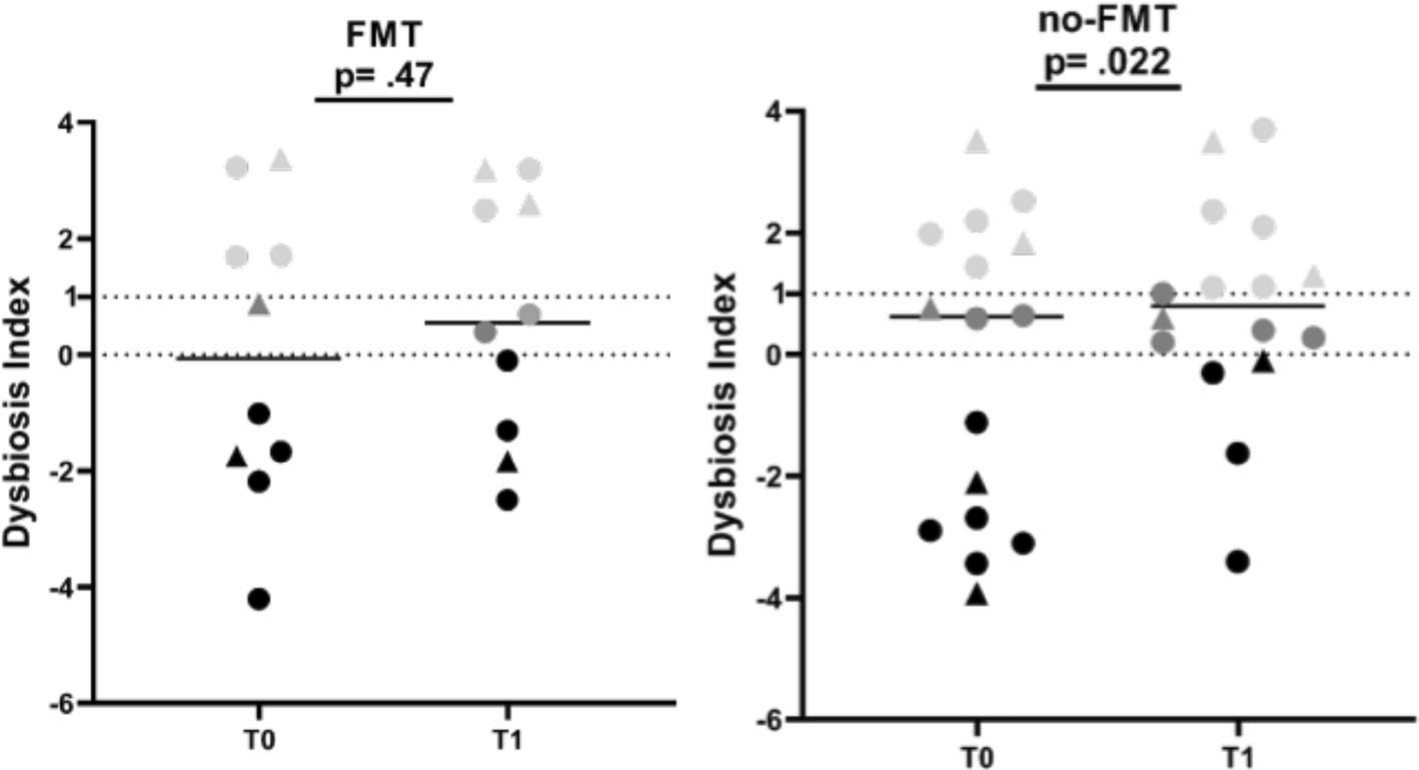
DI in cats in the FMT and in the non‐FMT group at T0 and at T1. No significant difference was found in the DI from T0 to T1 in either the FMT or the non‐FMT group. Black symbols represent healthy microbiota (DI < 0), gray symbols represent mild dysbiosis (DI 0–1), and light gray symbols represent severe dysbiosis (DI > 1). ⚫ Cats with diagnosis of CIE, ▲Cats with diagnosis of SCGL.

No significant difference of the DI was identified between T0 and T1 in either the FMT (mean 0.01, SD 2.5 vs. mean 0.7, SD 2.1; *p* = 0.47) or the non‐FMT group (mean −0.2, SD 2.5 vs. mean 0.7, SD 1.7; *p* = 0.09) (Figure 3). No significant difference was found in the abundance of *C. hiranonsis* between T0 and T1 in the FMT group (median 6.3, IQR 4.9–6.6 vs. median 5.4, IQR 4.1–6.0; *p* = 0.37) (Figure 3). No significant difference was found in DI between the FMT group and the non‐FMT group at either T0 (mean −0.2, SD 2.5 vs. mean −0.4, SD 2.4; *p* = 0.86) or at T1 (mean 0.7, SD 2.1 vs. mean 0.8, SD 1.8; *p* = 0.92) (Figure 3). No significant difference was found in the abundance of *C. hiranonsis* between the FMT group and the non‐FMT group at either T0 (median 6.3, IQR 5.9–6.6 vs. median 6, IQR 5.4–6.8; *p* = 0.99) or at T1 (median 5.4, IQR 4.1–6.0 vs. median 5.13, IQR 2.8–6.2; *p* = 0.85) (Figure 3). In the FMT group, 5/10 cats had an increased DI (> 0) at T0 and remained increased in 4 cats after FMT. In 2 of 5 cats with normal DI, the DI increased after FMT. In the non‐FMT group, 9/16 cats had an increased DI at T0, which remained increased in all at T1, whereas in 3/16 cats with normal DI at T0 the DI increased at T1.

### 3.7 FCEAI in Cats With CE Before and After FMT

**FIGURE 4:**
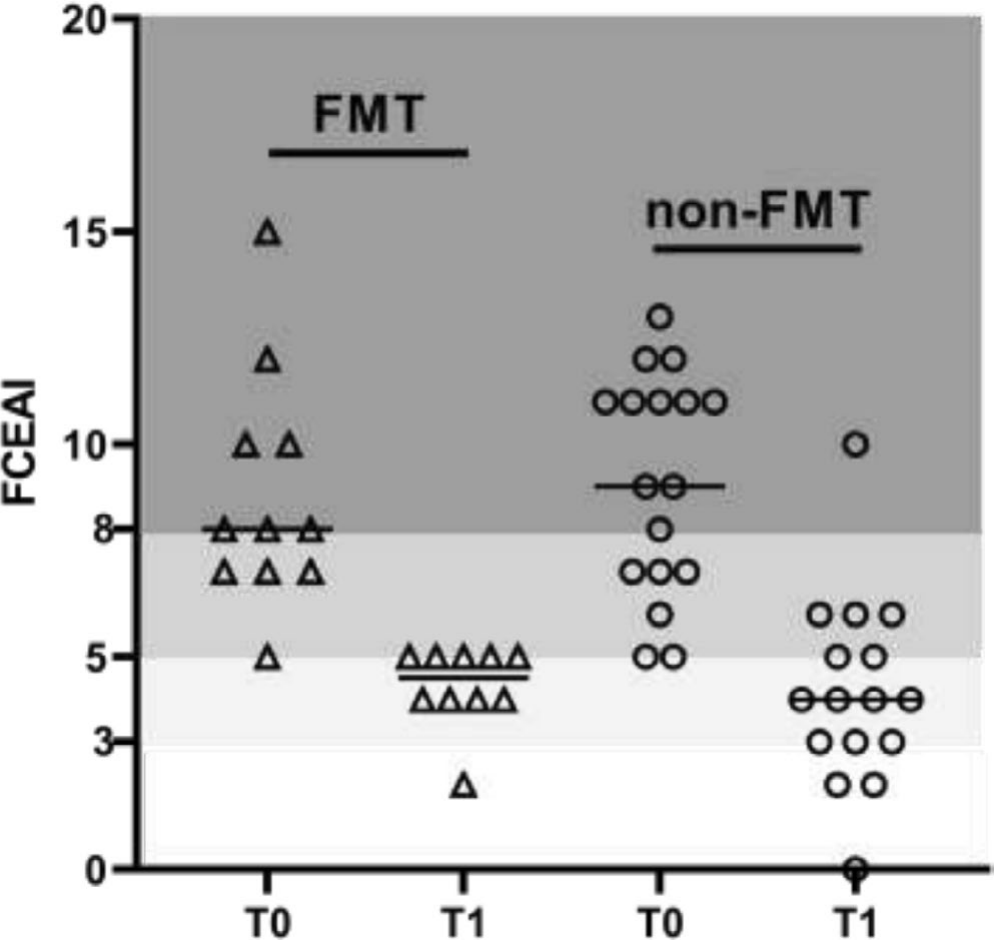
FCEAI at T0 and at T1 in cats in the FMT and the non‐FMT group. No significant difference was found in FCEAI between the FMT and the non‐FMT group at T0 or T1. О non‐FMT group, △ FMT group. Darker colors represent higher severity levels of FCEAI.

The FCEAI significantly decreased in cats in the FMT group 30 days after FMT (median 10.0, IQR 7.7–11.3 vs. median 4.5, IQR 4.0–5.0; *p* = 0.02) (Figure 4). The FCEAI also significantly decreased in cats in the non‐FMT group (median 9.0, IQR 7.0–11.0 vs. median 3.5, IQR 3.0–5.8; *p* < 0.001) by day 30 (Figure 4). However, there was no significant difference in the FCEAI between the FMT group and the non‐FMT group at either T0 (median 9.0, IQR 8.0–11.0 vs. median 9.0, IQR 7.0–11.0; *p* = 0.68) or T1 (median 4.5, IQR 4.0–5.0 vs. median 4.0, IQR 3.0–5.8; *p* = 0.63). None of the cats had increased FCEAI at T1 compared to T0.

## 4 Discussion

This is a prospective, blinded, controlled study evaluating the efficacy of single enema FMT as an adjunct tool in the management of CE in cats. Our study using this FMT protocol failed to show any differences between the FMT group and non‐FMT group of cats with regard to changes in fecal dysbiosis (as assessed by DI) [10] or the clinical disease activity score (FCEAI) [29].

CE is commonly seen in cats and can be associated with a high morbidity and death rate. Until recently, treatment of these cats was based on therapeutic trials with diet or immunosuppressive drugs or a combination of these, whereas the efficacy of antibiotics in cats with CE is doubtful to some clinicians [12]. Therapeutic strategies targeting the GI microbiota, such as those utilizing prebiotics, probiotics, or a combination of both (synbiotics), have been used in cats with CE [35], mainly as an adjustment treatment. The gut microbiota is a complex ecosystem and is composed of bacteria, archaea, fungi, protozoa, yeast, viruses, and parasites, although its composition is difficult to describe in its entirety. Thus, a more holistic intervention, such as FMT, may be more efficacious than probiotics, prebiotics, or synbiotics. FMT, a non‐pharmacological experimental treatment, which aims to restore intestinal microbial diversity and richness to a normal functional status, has gained popularity in recent years in both human and veterinary medicine [17, 19, 21, 22, 23, 24, 26, 36, 37].

In humans, cats, and dogs, CIE is considered a multifactorial disorder, and the interplay between the host immune system, inflammation, genetic factors, and the intestinal microbiota is believed to be involved in disease development [24, 38, 39]. Studies suggest that GI dysbiosis influences the pathogenesis and progression of GI diseases in cats and dogs [7]. In cats, dysbiosis is associated with decreased colonization resistance [40]. In one study, all antibiotic‐treated cats developed clinical signs after experimental infection with enteropathogenic *E. coli*, and administration of a probiotic containing *Enterococcus hirae* decreased the severity of clinical signs [40]. In contrast, cats not exposed to antibiotics that had a healthy microbiome were resistant to infection [40]. In one study in dogs, the interaction of macrophages collected from healthy dogs and bacteria isolated from CIE dogs resulted in a significantly greater phagocytosis and release of cytokines compared to those observed after the interaction of healthy macrophages and bacteria isolated from healthy dogs. According to this study, the GI microbiota of dogs with CIE may be genetically defective, triggering GI inflammation [41].

In our study, changes in the gut microbiota, as expressed by an increased DI, were noted in some cats with CE, with half of the cats with CE having an increased DI. Moreover, in our study, cats with SCGL had a significantly lower abundance of *C. hiranonsis* than cats with CIE; 33% of the cats with SCGL and 10.5% of the cats with CIE had decreased abundance of *C*. *hiranonsis*. While a previous study in cats failed to find a significant difference in the targeted bacterial taxa between cats with CIE and SCGL, a subset of cats with CE also had a decreased abundance of *C. hiranonsis*, which is also associated with a decreased ability of conversion of primary to secondary bile acids [10]. These findings support the theory that the microbiota is, at least in part, involved in the pathogenesis and/or progression of CE in cats. However, in our study, the DI did not significantly improve after FMT. In two cats, the DI increased at 30 days after FMT. We cannot be sure why this happened, but the microbiome may be, to some degree, variable in this disease, or it progressed despite some clinical improvement. However, it seems unlikely that the FMT alone caused an increase in DI.

Although no significant difference was found in DI, there was a significant clinical improvement in cats that received FMT, and FCEAI was decreased in all cats in the FMT group after 30 days. We are not able to ensure that the decreased FCEAI was due to FMT, as cats also underwent a diet change and received immunosuppressive medications, such as prednisolone or prednisolone in combination with chlorambucil. It would have been preferable if the cats that received FMT had not received any additional treatment, but the purpose of this study was to evaluate FMT as an adjunct treatment and not as monotherapy. In addition, because the benefits from FMT in cats with CE are unproven, it would be unethical to use FMT as a sole treatment. When we compared the FCEAI in cats in the FMT group with the FCEAI in cats in the non‐FMT group at day 30, no significant difference was found. Based on these results, FMT, administered only once, does not seem to be beneficial as an adjunct treatment in the cats in our study. In a preliminary study of 13 dogs with CIE that received corticosteroid therapy and a hypoallergenic diet and were randomized to receive either placebo or one single FMT, the addition of FMT to the treatment protocol also failed to show any difference in clinical scores after 30 days [36]. In a recent retrospective study, in 41 dogs with CE refractory to standard treatment, the DI at baseline was significantly lower for good FMT‐responders versus poor FMT‐responders, and dogs with a more severe dysbiosis responded more poorly to FMT compared to those dogs with a milder form of dysbiosis [37]. These results suggest that while FMT can be useful as an adjunctive therapy in dogs with CE and a mild form of dysbiosis, more severe dysbiosis may require more aggressive and repeated therapy, similar to what has been shown in humans [37], although in our study, the 1 cat with a mildly increased DI showed a severely increased DI after FMT administration.

There are several potential explanations for the lack of significant effect of FMT in the treatment of cats with CE. First, since the design of this study, clinical experience in humans and dogs has suggested that multiple doses of FMT are likely needed in many cases [23, 42]. Second, our study evaluated the effect of FMT 30 days after a single FMT administration. It is plausible that FMT might have transiently improved dysbiosis, but by the time DI and FCEAI were evaluated, this effect was no longer present. This is in contrast to initial experiences with FMT for antibiotic‐induced dysbiosis. For example, in one pilot study, the feline DI recovered significantly quicker in cats that received one single FMT after a 10‐day course of amoxicillin and clavulanate when compared with cats in the control group [43]. It is possible that in cats with CE with more severe or permanent lesions in the intestinal mucosa, repeated FMT would be needed to treat dysbiosis more definitively in cats with CE. Therefore, further studies using different FMT protocols and more intense evaluations of the outcome are warranted before FMT can be considered ineffective as an adjunct tool in the management of cats with CE. For example, in one case report in a cat with CIE, which responded well to the first FMT, the FMT enema needed to be repeated at week 5 for long‐term improvement [43]. Furthermore, this is in line with studies in dogs. In one pilot study in 3 dogs with CE, the canine DI was performed every week for 2 months after a single FMT [37]. The DI decreased within 1 week after FMT but started to increase again after 3–4 weeks. Similarly, results (unpublished) were shown in an ACVIM abstract, where a significant decrease in the DI was seen 1 week after FMT in 16 dogs with chronic diarrhea, but 4 weeks after the single FMT, the DI increased again in some dogs [44].

When our study was designed, no evidence‐based FMT protocols had been established for use in cats and dogs [44]. In our study, we used a single FMT course administered via enema based on a previous case report in cats, in previous reports in dogs, and in unpublished reports [43, 45, 46]. Our target was to make this process as simple as possible and easy to perform in clinical practice, and something that the owners would agree to do. Our dose may have been too low, as most recent studies and reports using FMT successfully in dogs reported a dose between 5 and 7 g/kg BW of FMT vs. our dose of 2 g/kg BW [42, 47]. Another possibility why our study failed to show significant improvement after FMT is the choice of the route of administration. However, a previous study in a cat reported a significant improvement after a fecal enema, with a repeated enema 5 weeks later [46]. Also, studies in dogs revealed that FMT treatment was both effective via oral or rectal application [47, 48, 49, 50, 51]. To the authors' knowledge, there are no studies comparing the administration of FMT via enema with the other routes of administration in cats. More studies involving larger numbers of animals and more defined phenotypes of intestinal disease are needed to further evaluate the efficacy of different administration routes.

Our study had some limitations. One limitation is the relatively small number of cats in each group. A post hoc power analysis was conducted to assess the statistical power of the study regarding the differences between the FMT group and the non‐FMT group. The analysis revealed that the study was underpowered for both primary outcomes; the power for FCEAI was 0.05, and for the DI was 0.24. This low power suggests a high risk for a Type II error, meaning that the study may have been insufficiently sensitive to detect a significant difference between the groups, even if one exists. Our study was a prospective study, with relatively strict inclusion and exclusion criteria, and therefore it is challenging to enroll large numbers of animals. Another limitation could be potentially the enrollment of cats with concurrent diseases, and this may have affected the clinical response of some of the cats. In the authors' institutions, many (if not the majority) of cats with CE present with one or more concurrent diseases, mainly pancreatitis, kidney disease, and others. Therefore, we feel that the feline sample enrolled in our study reflects what is commonly encountered in clinical practice. Concurrent diseases such as CKD could also impact DI [52]. Two cats with CIE in our study (both in the non‐FMT group) had CKD stage 2, and compared to healthy cats (≥ 8 years), cats with CKD have been documented to have a dysbiosis characterized by decreased fecal microbial diversity and richness [52].

Another limitation of the current study is that the cats not only received FMT at T0 but were also switched to another diet. This was done for two reasons: (a) to test for FRE that have not responded to previous diet changes and (b) so that the cats were all on the same diet during the study, thus minimizing differences in the microbiota that could be attributed to diet. However, several of the cats refused to consume the diet. In addition, when the histopathological diagnosis was available, cats received various medications as their main treatment, including prednisolone and chlorambucil. These additional treatments were not consistent between cats, as some of the cats refused to consume the hydrolyzed diet and others did not respond to diet alone. Clearly, this was not ideal, but it would be considered unethical not to treat cats with lymphoma or severe IBD with standard treatment. Additionally, as mentioned above, the purpose of this study was to evaluate FMT as an adjunct treatment and not as monotherapy. Lastly, in our study, cats with previous antibiotic administration were also enrolled, and this could also influence the results due to antibiotic‐induced dysbiosis. Although 3 weeks might be considered a short timeframe for the impact of antibiotics in the microbiota, FMT is reported to improve also antibiotic‐induced dysbiosis. In clinical practice, in such chronic diseases, many cats have already received various antibiotic treatments that can cause or further exacerbate an already disturbed microbiota, so a treatment such as FMT is needed also in these situations.

In conclusion, in our study of cats with CE, a single enema FMT did not lead to a significantly different clinical or DI improvement compared to cats with that did not receive FMT. A benefit of FMT in CE cats using a different FMT protocol cannot be excluded based on our results. The procedure was well tolerated and no adverse effects were noticed, none of the cats of our study that received FMT showed any aggravation of the signs of GI disease.

## Disclosure

Authors declare no off‐label use of antimicrobials.

## Ethics Statement

The study protocol was reviewed and approved by the Animal Ethics Committee of the University of Thessaly, Greece (AUP number:115/6.10.2020). Authors declare human ethics approval was not needed.

## Conflicts of Interest

Jan S. Suchodolski, Joerg M. Steiner, and Jonathan A. Lidbury are employees of the Gastrointestinal Laboratory at Texas A&M University, which offers gastrointestinal function testing on a fee‐for‐service basis.

## Supporting information


Data S1.
