# Supplementary material for: Single Enema Fecal Microbiota Transplantation in Cats With Chronic Enteropathy
Source: J Vet Intern Med. 2025 Apr 10;39(3):e70054. doi: 10.1111/jvim.70054 (PMC11983779; doi:10.1111/jvim.70054)
Supplement: Supplementary file 1 — Data S1. [file JVIM-39-e70054-s001.docx]

*Dysbiosis Index in cats with CE before and after FMT using fold change comparison*

Based on the fold change comparison, the FMT group showed a 0.04-fold lower DI on day 0 compared to the non-FMT group, indicating that cats in the FMT group had 94% lower DI at baseline compared to the cats in non-FMT group. The corresponding log_2_ fold change of -4.64 suggests a negative shift in the FMT group, with an approximate 0.04-times lower DI in FMT-group at baseline compared to non-FMT group (Table 4). At T1 (30 days after the administration of FMT) the FMT group showed a 0.87-fold decrease in DI relative to the non-FMT group, indicating that the FMT led to an approximately 13% decrease in DI compared to the non-FMT group. The corresponding log_2_ fold change of -0.2 suggests a negative shift in the FMT group, with an approximate 1.14-times lower DI compared to the non-FMT group at T1 (Table 4).

*FCEAI in cats with CE before and after FMT using fold change comparison*

Based on the fold change comparison, the FMT group had a 1.09-fold higher FCEAI on day 0 compared to the non-FMT group, indicating that cats in the FMT group had 9% higher FCEAI at baseline compared to the cats in non-FMT group. The corresponding log_2_ fold change of 0.12 suggests a positive shift in the FMT-group, with an approximate 1.09-times higher FCEAI in FMT-group at baseline compared to non-FMT group (Table 4). At T1 (30 days after the administration of FMT) the FMT group showed a 1.01-fold increase in FCEAI relative to the non-FMT group, indicating that the FMT led to an approximately 1% increase in FCEAI compared to the non-FMT group. The corresponding log_2_ fold change of 0.01 suggests a positive shift in the FMT group, with an approximate 1.01-times higher FCEAI in FMT-group at T1 compared to non-FMT group (Table 4).

*C. hiranonsis in cats with CE before and after FMT using fold change comparison*

Based on the fold change comparison, the FMT group had a 0.99-fold lower abundance of *C. hiranonsis* at day 0 compared to the non-FMT group, indicating that cats in the FMT group had 0.1% lower abundance of *C. hiranonsis* at baseline compared to the cats in non-FMT group. The corresponding log_2_ fold change of -0.02 suggests a negative shift in the FMT-group, with an approximate 0.99-times lower abundance of *C. hiranonsis* in FMT-group at baseline compared to non-FMT group (Table 4). At T1 (30 days after the administration of FMT) the FMT group showed a 1.08-fold increase in abundance of *C. hiranonsis* relative to the non-FMT group, indicating that the FMT led to an approximately 8% increase in abundance of *C. hiranonsis* compared to the non-FMT group. The corresponding log_2_ fold change of 0.11 suggests a positive shift in the FMT-group, with an approximate 1.08-times higher abundance of *C. hiranonsis* in FMT-group at T1 compared to non-FMT group (Table 4).

**Table 4**

Fold change comparisons of DI, FCEAI and *C.hiranonsis* at baseline and 30 days after FMT between cats that received FMT (FMT-group) and the control group of cats (non-FMT group).

| Measurement | FMT group mean | non-FMT group mean | Fold change | log_2_ \|Fold change\| |
| --- | --- | --- | --- | --- |
| DI Day 0 | 0.01 | -0.23 | -0.04 | -4.64 |
| DI Day 30 | 0.7 | 0.8 | 0.87 | -0,2 |
| FCEAI Day 0 | 9.7 | 8.94 | 1.09 | 0.12 |
| FCEAI Day 30 | 4.3 | 4.25 | 1.01 | 0.01 |
| *C. hiranonsis* Day 0 | 5.26 | 5.31 | 0.99 | -0.02 |
| *C. hiranonsis* Day 30 | 4.85 | 4.5 | 1.08 | 0.11 |
